# Efficacy and safety of Xiaoyao pills for mild to moderate depression: study protocol for a randomized controlled trial

**DOI:** 10.1186/s13063-021-05909-y

**Published:** 2022-01-04

**Authors:** Zhe Xue, Zhen Huang, Shu-li Cheng, Xi-hong Wang, Xuan Zhou, Qing-yu Ma, Jia-xu Chen

**Affiliations:** 1 School of Traditional Chinese Medicine, Beijing University of Chinese Medicine, No. 11, Bei San Huan Dong Lu, Chaoyang District, Beijing, 100029 China; 2 Guangzhou Key Laboratory of Formula-Pattern of Traditional Chinese Medicine, School of Traditional Chinese Medicine, Jinan University, No. 601 Huangpu Avenue West, Guangzhou, 510630 China

**Keywords:** Traditional Chinese Medicine, Depression, Randomized controlled trial

## Abstract

**Background:**

Depression is one of the most frequent and severe psychiatric conditions. Many chemical drugs to treat depression are associated with adverse reactions and have shortcomings. Traditional Chinese medicine is of great significance in the prevention and treatment of depression. Xiaoyao pills has achieved good results in clinical application, which has the advantages of quick effect and no obvious adverse reactions. The aim of our study is to evaluate the efficacy and safety of Xiaoyao pills on mild to moderate depression patients.

**Methods:**

This study is a multi-centre, double-blinded, randomized and placebo-controlled clinical trial. A total of 108 participants are assigned to three groups: Xiaoyao pill group taking Xiaoyao pills twice daily for 4 weeks, placebo group taking placebos twice daily for 4 weeks and normal group without taking any drug. The primary and secondary outcome measures are the Hamilton Depression Scale (HAMD) and Traditional Chinese Medicine (TCM) Syndrome Scale. The assessment is at baseline (before treatment initiation), 1 week, 2 weeks 4 weeks after the first treatment. Exploratory outcome is also assessed to explore the mechanism of Xiaoyao pills at baseline and 4 weeks.

**Discussion:**

The results from this study will provide clinical evidence on the efficacy and safety of Xiaoyao pills in patients with mild to moderate depression with syndrome of liver stagnation and spleen deficiency.

**Trial registration:**

ClinicalTrials.gov ISRCTN12746343. Registered on September 25, 2020.

## Administrative information

**Table:** 

Title {1}	Efficacy and safety of Xiaoyao pills for mild to moderate depression: study protocol for a randomized controlled trial
Trial registration {2a and 2b}.	International Standard Randomized Controlled Trial Number Register: ISRCTN12746343.Registered on September 25, 2020. (http://www.isrctn.com/ISRCTN12746343)
Protocol version {3}	June 1, 2019, version 3
Funding {4}	The study was supported by Youth Science Fundation Project of the National Natural Science Foundation of China (Grant No. 81803972), Key Project of the National Natural Science Foundation of China(Grant No.81630104) and Huang Zhendong Research Fund for Traditional Chinese Medicine of Jinan University.
Author details {5a}	^1^School of Traditional Chinese Medicine, Beijing University of Chinese Medicine, Beijing, China, No. 11, Bei San Huan Dong Lu, Chaoyang District, Beijing 100029, China ^2^Guangzhou Key Laboratory of Formula-Pattern of Traditional Chinese Medicine, School of Traditional Chinese Medicine, Jinan University, Guangzhou, Guangdong, China, No. 601 Huangpu Avenue West, Guangzhou, 510630, China
Name and contact information for the trial sponsor {5b}	School of Traditional Chinese Medicine, Beijing University of Chinese Medicine Address: No. 11, Bei San Huan Dong Lu, Chaoyang District, Beijing 100029, China Website: http://www.bucm.edu.cn/
Role of sponsor {5c}	The role of the funding body: the National Natural Science Foundation of China (NSFC) is a funding institution, which is responsible for providing funds and supervising whether the research is completed. The Huang Zhendong Research Fund also provides part of funds. The role of the sponsor: Beijing University of Chinese Medicine is the sponsor, which is responsible for the study design; collection, management, analysis, and interpretation of data; writing the report; and the decision to submit a report for publication; and Beijing University of Chinese Medicine has the final power to interpret these activities.

## Introduction

### Background and rationale {6a}

Depression is one of the most frequent and severe psychiatric conditions, with an estimated prevalence reaching 15% in the general population [1]. Depression is characterized by sadness, loss of interest and pleasure, feelings of guilt, feeling of worthlessness, low appetite, fatigue, and poor concentration [2]. Depression is known to dramatically increase the risk of premature death by suicide or other general medical conditions, such as vascular diseases [1]. Considering the alarming impact of COVID-19 infection on mental health [3, 4], we should pay more attention to the treatment of depression. There are many treatments, including pharmacological, psychological, and neurostimulatory options that are associated with remission of symptoms and full restoration of psychosocial function. Unfortunately, a significant proportion of patients do not achieve sustained remission, despite serial treatments [5]. Clinical practice has proven that many chemical drugs have beneficial therapeutic effects in depression, but they are also associated with adverse reactions, such as drowsiness and dystonia, and have shortcomings, such as a narrow antidepressant spectrum and a high rate of relapse [6].

Traditional Chinese medicine (TCM) is of great significance in the prevention and treatment of depression. Xiaoyao pills, which is one of traditional Chinese medicine prescription, have been used for both the prevention and treatment of chronic dizziness for thousands of years. It has achieved good results in clinical application, which has the advantages of quick effect and no obvious adverse reactions [7]. Xiaoyao pills consist of Radix Bupleuri (root of Bupleurum Chinense DC.), Radix Paeoniae Alba (root of Paeonia lactiflora Pall.), Radix Angelicae Sinensis (root of Angelica sinensis (Oliv.) Diels), Rhizoma Atractylodis (root and rhizome of Atractylodes lancea (Thunb.) DC.), Poria (fungus nucleus of Poria cocos (Schw.) Wolf), Radix Glycyrrhizae (root and rhizome of Glycyrrhiza uralensis Fisch.), Herba Menthae (aboveground portions of Mentha haplocalyx Briq.), and Rhizoma Zingiberis Recens (fresh root and rhizome of Zingiber officinaleRosc.) in a ratio of 5:5:5:5:5:4:1:1 [8]. The antidepressant mechanism of Xiaoyao pills was widely studied from the molecular biological level recent years [9–11]. At the same time, there are many clinical reports of Xiaoyao pills [12–14]. However, the quality of most clinical report methods needs to be improved.

### Objectives {7}

This study intends to carry out a multi-center randomized controlled and double-blind trial to observe the efficacy of Xiaoyao pills in the treatment of mild to moderate depression.

### Trial design {8}

This study is a multi-center, double-blinded, randomized, placebo-controlled, parallel group and exploratory clinical trial with allocation radio 1:1. The trial protocol has been approved by the ethical committee of the Beijing University of Traditional Chinese Medicine (ref: 2020BZYLL0304) and is registered with ISRCTN at Current Controlled Trials (ISRCTN 12746343). The patients are provided with written informed consent. The trial is based on the principles of ICH-GCP [15] and appropriate legal regulations for recommended items to address in a clinical trial protocol according to the SPIRIT 2013 Checklist [16] and the Consolidated Standards of Reporting Trials (CONSORT) statement [17].

## Methods: participants, interventions, and outcomes

### Study setting {9}

#### Study setting and participants

**Fig. 1:**
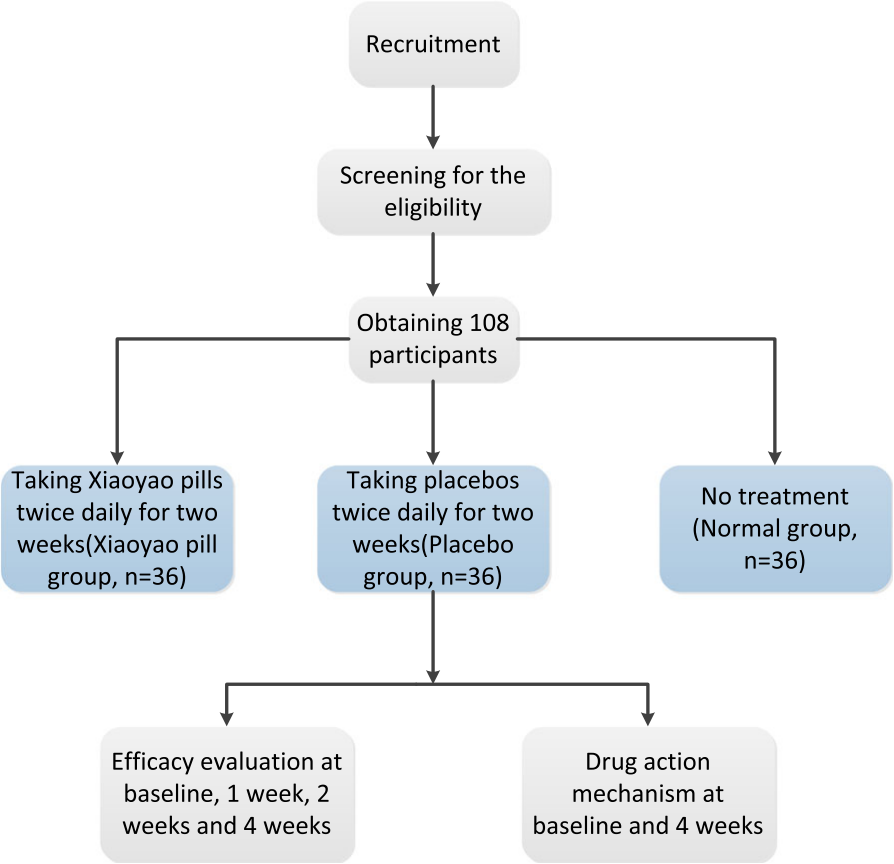
Study sequence

A total number of 72 participants with mild-to-moderate depression (referred to symptoms of liver stagnation and spleen deficiency) and 36 healthy participants are recruited at the five trial sites in china, including Peking University Sixth Hospital, the First Affiliated Hospital of Jinan University (Guangzhou Overseas Chinese Hospital), the Affiliated Brain Hospital of Guangzhou Medical University, Dongfang Hospital (Beijing University of Chinese Medicine Second Affiliated Hospital), and Beijing Anding Hospital Capital Medical University. Participants are informed of details about the study which are purpose, duration, procedures, and key contacts, as well as risks and potential benefits. Participants may withdraw their consent for any reason without any consequences at any time. Eligible 72 participants with mild-to-moderate depression are randomly allocated to Xiaoyao pill group (Group 1) and placebo group (Group 2), who are required to take Xiaoyao pills and placebos, respectively, twice daily for four consecutive weeks. 36 healthy participants are allocated to the normal group (Group 3) without taking any drug. The flow chart is listed in Fig. 1.

### Eligibility criteria {10}

#### Inclusion criteria

Participants meeting the following criteria are included:

1. Meet the Diagnostic Statistics Manual of Mental Disorders (DSM-5) regarding the diagnosis of mild to moderate depression;

2. A score between 20 and 35 on Hamilton Depression Scale (HAMD);

3. Meet the TCM criteria of liver stagnation and spleen deficiency syndrome;

4. Aged between 16 and 18 years old;

5. Patients agree to participate in this trial and assign the informed consent;

6. Capable of reading and follow-up treatment, and permanently live locally.

#### Exclusion criteria

Participants meeting one or more of the following criteria are excluded:

1. Bipolar depression, treatment-resistant depression, and severe suicidal risk;

2. History of bipolar disorder, schizophrenia, obvious psychotic symptoms, and depression disorder caused by non-addictive substances;

3. Combine with severe cardiovascular diseases, cerebrovascular diseases, hepatic diseases, renal diseases, hematological disease, cancer, or other severe primary diseases;

4. Pregnant or lactating women;

5. Inability to finish the compliance test, judge the efficacy, and have complete data;

6. Involved with any other clinical trial at the time of consent.

#### Who will take informed consent? {26a}

All participants with depression enrolled in the study as well as 36 healthy people sign a paper version of informed consent prior to study initiation. The recruitment of participants in each study centre is made by the researchers (doctors) in each study center. Researchers make face-to-face evaluation of patients or healthy people who are willing to participate in the project. If the participants meet inclusion criteria, the researchers inform them to sign the informed consent. Only after signing the informed consent can the trial process be officially started. Two copies of the informed consent were kept by the researchers and the participants, respectively.

#### Additional consent provisions for collection and use of participant data and biological specimens {26b}

Standard clinical laboratory findings (including complete the blood count, liver, and kidney function tests) as indicators of safety evaluation are routinely collected before and after the enrollment of patients to ensure the safety of subjects. Participants’ urine samples and blood samples are monitored by liquid chromatography-mass spectrometry (LC-MS) technology to explore the mechanism of action of the metyrapone at baseline and at 4 weeks. At the same time, participants’ blood samples are used to separate the total RNA. The study participants have given consent for their blood samples and data to be used. The ethics committee has given consent to the trialists to conduct the study. All the samples required for the study do not need to sign additional informed consent, because the committee has already been approved by the ethics committee and can use the data after signing the informed consent.

### Interventions

#### Explanation for the choice of comparators {6b}

The placebos were manufactured by the Jiuzhitang Co., Ltd. Placebos are similar to the physical traits of Xiaoyao pills in size, flavor, scent, and color without the key ingredients. The placebos are composed of corn starch, pregelatinized starch, maltose, caramel color, and water.

### Intervention description {11a}

Participants randomized to treatment Group 1 take Xiaoyao pills, which are composed of ChaiHu (Radix Bupleuri)-100g, DangGui (Radix Angelicae Sinensis)-100g, BaiShao (Radix Paeoniae Alba)-100g, BoHe (Mentha)-20g, ShengJiang (Ginger)-100g, BaiZhu (Atractylodes macrocephala Koidz) - 100 g, FuLing (Poria cocos Wolf) -100g, and ZhiGanCao (Glycyrrhiza uralensis Fisch)-80g. The action and batch number of each herb are summarized in Table 1. Xiaoyao pills are water honey pills (Z20013060) manufactured by the Jiuzhitang Co., Ltd. Participants in Group 2 take placebos which are manufactured by the Jiuzhitang Co., Ltd. Placebos are similar to the physical traits of Xiaoyao pills in size, flavor, scent, and color without the key ingredients. The placebos are composed of corn starch, pregelatinized starch, maltose, caramel color, and water. Participants are required to take the medicine twice daily for four consecutive weeks and the dosage is 9g twice a day. The normal group are not given any drug.

**Table 1:** The action and batch number of each herb

Ingredients	Lot number	Action
***BaiZhu*** ***(Atractylodes*** ***Macrocephala Koidz)***	018AJ-2005005-725	TCM: Tonifying *Qi* of the spleen, eliminating dampness, and diuresis.
***Chaihu*** ***(Radix Bupleuri)***	FM201538	TCM: Soothing the liver and tonifying qi, relieving depression, and reducing fever. Pharmaceutical study: (1) sedative effect. (2) analgesic effect. (3) Anti-pathogen effect.
***Danggui*** ***(Radix Angelicae Sinensis)***	FM201541	TCM: Moisturizing the intestines promoting blood circulation and removing blood stasis, regulating menstruation, and relieving pain.
***Baishao*** ***(Radix Paeoniae alba)***	FM201030 FM201037 FM201041	TCM: Restraining yin and stopping sweating, nourishing blood and regulating menstruation, calming liver yang, softening liver, and relieving pain.
***Fuling*** ***(Poria cocos Wolf)***	FM201540	TCM: Diuresis, detumescence, tonifying *Qi* of the spleen, and tranquilization. Pharmaceutical study: (1) Diuresis effect. (2) Calming effect. (3) Anti-tumor effect. (4) Anti-diabetic effect. (5) Enhance myocardial contractility.
***Zhigancao*** ***(Glycyrrhiza uralensis Fisch)***	FM200983	TCM: Tonifying *Qi* of the spleen, eliminating phlegm, anti-cough, heat-clearing and detoxifying, and reconciling. Pharmaceutical study: (1) Anti-arrhythmia effect. (2) Anti-ulcer effect. (3) Diuresis effect. (4) Analgesia effect. (5) Anti-tussive effect. (6) Eliminating phlegm effect.
***Bohe*** ***(Mentha)***	FM201542	TCM: Evacuating wind-heat, clearing the boss, relieving the throat, clearing the rash, soothing the liver, and promoting qi. Pharmaceutical study: (1) Anti-inflammatory effect. (2) Sedative effect. (3) Choleretic effect. (4) Penetration promoting effect.
***Shengjiang*** ***(Ginger)***	FM201539	TCM: Relieving the appearance and dispelling cold, warming the middle to stop vomiting, warming the lungs and detoxifying. Pharmaceutical study: (1) Antibacterial effect. (2) Anti-cancer effect. (3) Anti-oxidation effect. (4) Anti-aging effects.

### Criteria for discontinuing or modifying allocated interventions {11b}

#### Shedding criteria

Cases that have been enrolled but do not complete the clinical protocol should be considered drop outs in the following circumstances:

1. Patients withdrew from the trial on their own;

2. Lost to follow-up;

3. Poor compliance;

4. Because some diseases are considered by the study physician to be amenable to withdrawal from the trial.

The case should be explained. If the baseline pharmacodynamic data are available, the results of the last major outcome can be transferred to the final result for statistical analysis, and the research records should be kept for reference.

#### Discontinuing criteria

The entire trial is completely stopped in multi-centers for the following reasons:

1. Serious safety concerns identified by the investigator;

2. There are major lapses in the trial;

3. Reasons for funding or management by the sponsor;

4. The administrative department cancels the trial.

Total discontinuation of the trial can be temporary or permanent. When discontinuing a trial, full trial records should be retained.

#### Rejection criteria

Cases that have been enrolled but meet one of the following should be removed:

1. Cases were incorrectly diagnosed and incorrectly included;

2. Met the exclusion criteria;

3. One medication was not used;

4. Without any test record;

5. Due to the use of some prohibited medication, it was not possible to evaluate drug efficacy.

Rejected cases should state the reason, and their original medical records should be retained for statistical analysis of efficacy.

#### Strategies to improve adherence to interventions {11c}

Clinical research coordinator is set up to guarantee subject compliance. Drug record cards are also distributed to allow patients to record their medication status and follow-up time.

#### Relevant concomitant care permitted or prohibited during the trial {11d}

##### Concomitant treatments and forbidden drugs

1. Avoid cold and greasy foods that are difficult to digest.

2. During the period of taking the medicine, keep optimistic, and avoid getting angry.

3. People with severe chronic diseases such as high blood pressure, heart disease, liver disease, diabetes, and kidney disease should take it under the guidance of a physician.

4. Normal menstruation, sudden excessive menstrual flow, prolonged menstrual period, or oligomenorrhea, wrong menstrual period, or irregular vaginal bleeding should go to the hospital for treatment.

5. People who are allergic to this product should not use it with caution.

6. It is forbidden to use this product when its properties change, such as from solid to liquid or packing damage.

7. If you are using other drugs, please consult your physician or pharmacist before using this product.

##### Provisions for post-trial care {30}

There are no expected harms resulting from the trial. After the trial, patients will be provided with guidance on specialized treatment protocols in psychiatry.

### Outcomes {12}

#### Primary outcome measures

##### Hamilton Depression Scale (HAMD)

The HAMD is a tool developed for clinical evaluation of depression and is widely used in studies [18]. It is composed of 17 questions. Each question is scored from 1 to 4 points, where a higher score indicates severe symptoms; 0 to 7 point is normal, 7 to 17 points may have depression, 17 to 24 points definitely have depression, and more than 24 points means severe depression. Measurements are taken at the baseline, 1 week, 2 weeks, and 4 weeks after treatment.

### Secondary outcome measures

#### Traditional Chinese Medicine (TCM) Syndrome Scale

TCM Syndrome Scale is used as a measure of depression [19]. It consists of 1 primary symptom and 9 second diagnostic symptoms. The participants are required with mental depression, and need to have more than 4 other concurrent symptoms of the main symptoms at the same time, and the symptoms should last for a 4-week session. Moreover, participants are provided with symptoms of stagnation of liver qi and spleen deficiency as follows: (1) suspicious; (2) fullness of chest and thigh; (3) chest tightness; (4) easy to sigh; (5) complexion is chlorosis; (6) stomach fullness; (7) abdominal pain; (8) bloating; (9) nausea; (10) bowel; (11) loose stools; (12) foreign body sensation in the pharynx; (13) light tongue, white tongue coating; and (14) pulse string is thin or slippery. Participant who is involved with more than 5 symptoms can be diagnosed. Measurements are taken at the baseline, 1 week, 2 weeks, and 4 weeks after treatment.

#### Exploratory outcome

The urine and blood of participants are monitored to explore the mechanism of Xiaoyao pills at baseline and 4 weeks by liquid chromatograph-mass spectrometer (LC-MS) technology. Meanwhile, the total RNA is isolated from the blood sample using TRIzol (Life Technologies). Differential expression is analyzed by using the TopHat method. Gene Ontology (GO) and Kyoto Encyclopedia of Genes and Genomes (KEGG) enrichment analysis of differentially expressed genes are implemented by the Methylmion Specific PCR. And the exploratory outcomes are expected by the network pharmacology to conduct the correlation analysis with the differential genes.

#### Participant timeline {13}

**Fig. 2:**
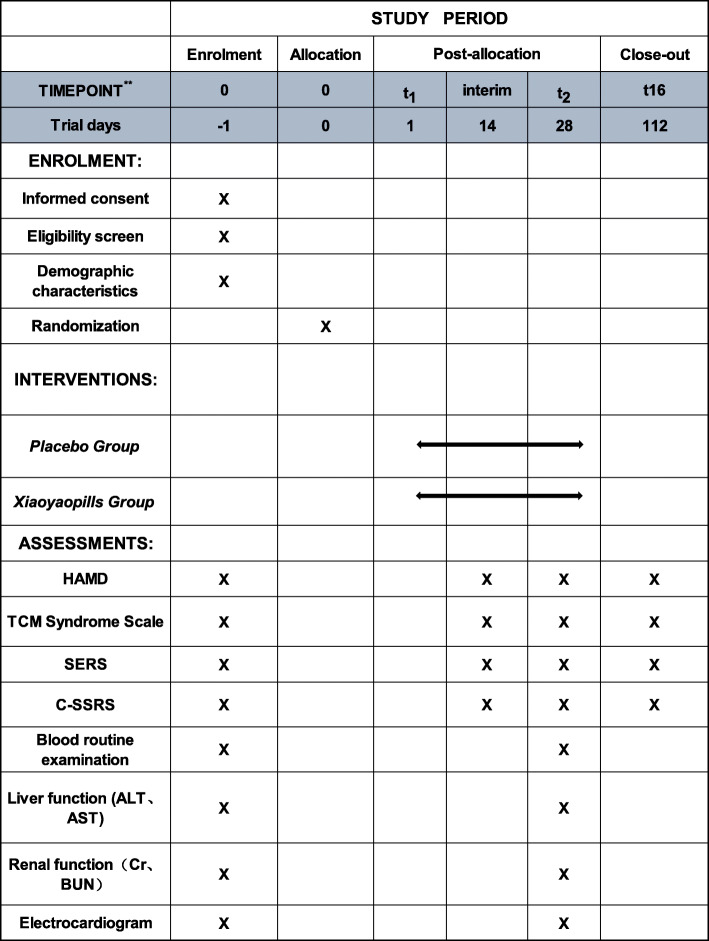
Schedule of enrolment, interventions, and assessments

Participant timeline is shown in Fig. 2.

#### Sample size {14}

Based on previous findings of Xiaoyao pills in the treatment of depression [20, 21], the efficacy rate of the control group and treatment group were 32.7% and 91.4%, respectively. According to rules of thumb, we use PASS software to calculate the sample size as 30 patients in the control group and 30 patients in the Xiaoyao Pills group in a ratio of 1:1 with a type I error (*α* = 0.05) and type II error (*β* = 0.1). Meanwhile, taking into account a dropout of 15%, 72 patients are recruited with another 36 healthy persons. Therefore, we calculated the sample size as 108 participants.

#### Recruitment {15}

A total number of 72 participants with mild-to-moderate depression (referred to symptoms of liver stagnation and spleen deficiency) and 36 healthy participants are recruited at the five trial sites in china. The number of registrations at each facility is monitored by the monitoring committee via electronic data capture (EDC). We publish clinical trial information through posters and WeChat public account articles. Posters are posted in various sub-centers and communities. WeChat public account articles are open to the general public. The contact information of the research center and the researchers is found in the posters and articles. At the same time, the researcher also recruits the participants in the outpatient clinic. They evaluate and recruit prospective candidates by introducing the trial to the them. The researcher conducts a face-to-face assessment of the potential participants. And the participants who meet the inclusion criteria are formally included in the clinical trial after signing the informed consent.

### Assignment of interventions: allocation

#### Sequence generation {16a}

The study adopts the method of central stratified block randomization. According to the given seed number and segment length, the random number table of 72 subjects is generated by an independent statistician using SAS 9.4 statistical software according to the ratio of 1:1 of the Xiaoyao pills group and the placebo group.

#### Concealment mechanism {16b}

In order to make the researchers and participants unable to predict the group of the next participant, we implement the allocation sequence with sequentially numbered, opaque, and sealed boxes. Drugs are put into medicine boxes of the same size and coded in sequence. The coding of all drugs is prepared and taped by a third-party person unrelated to the trial. After the participants are officially enrolled into the group, the researchers distributed the medicine boxes to the participants. Each study center cannot know participants’ information in other centers.

#### Implementation {16c}

The allocation sequence is generated and saved by statisticians who do not participate in the clinical study. The recruitment of participants is conducted by the researchers. Researchers assess whether the participants meet the inclusion criteria. After the participants who meet the inclusion criteria sign the informed consent form, the researchers send the sealed boxes containing drugs and coded in sequence to the participants and unable to know the specific intervention of the participants.

### Assignment of interventions: blinding

#### Who will be blinded {17a}

The study uses a double-blind design and drugs are coded and packaged blind according to a generated random number table by personnel unrelated to this trial. The drugs in each group are uniformly packaged, while guaranteeing that the Xiaoyao pills and placebos are not different in appearance. The centers dispense the drugs sequentially with the assigned drug number and in the order of subject enrollment. The participants, researchers are blinded to treatment allocation. All participants are informed that they have an equal opportunity for allocation to the Xiaoyao-pills group or placebos group before enrollment. Data analysts and outcome assessors do not participate in the blinding. Clinical trial related data, such as drug types and affiliated centers, is passed to data analysts and outcome assessors after uncovering blinding. The blind bottom is kept in duplicate by the principal investigator and the sponsor after sealing, and the blind bottom could not be removed during the trial.

#### Procedure for unblinding if needed {17b}

At the end of the trial, the first-level uncovering blinding is performed after checking CRF, EDC, and signature. The groups of subjects are determined by the first-level uncovering blinding to conduct statistical analysis. After the statistics analysis, the second-level uncovering blinding is performed. Through the second-level blinding, the Xiaoyao pill group and the placebo group are determined to evaluate the drug efficacy. Unblinding is generally done at the end of the trial for statistical analysis and outcome assessment. However, in order to ensure the safety of the subjects, the blindness is uncovered in some emergency situations, such as the occurrence of a serious adverse event, unable to judge its relationship with the trial drug, whether it is overdosed, whether it has serious drug interactions with the concomitant medication, etc. When it is urgent, researchers need to know what drug to take to decide the rescue regimen. After uncovering blindness, it is necessary to record the time, reason for breaking the blindness in advance and the person performing the blind breaking, and to notify the inspector and the sponsor as soon as possible. Once the blindness is uncovered in advance, the participants should not continue to participate in the study. The data of these participants cannot be used for the efficacy evaluation analysis, but the data should still be included in the safety analysis data set. And the participants should be treated and protected in time. An emergency letter is prepared for every participant with an envelope made of thick, light tight paper. Unblinding of a single participant should not disrupt the blinding of the entire clinical trial. Emergency letters are made by a third-party person unrelated to this trial.

### Data collection and management

#### Plans for assessment and collection of outcomes {18a}

The results and baseline of all participants are collected and evaluated on Electronic Data Capture System (EDC). Assessment and collection of outcome, baseline, and other trial data are performed and recorded on EDC. All data is collected and entered into the EDC system by researchers. The system has data entry range value and character. And the system preliminarily tests the rationality of the data. After the entry, data monitoring committee checks the data quality again. During the course of this study, the sponsor performs regular on-site monitoring visits to the study centers and trains researchers in every study centers to ensure that all the elements of the study protocol are strictly followed and that the data are completed properly. At the same time, a unified case report form, HDS and TCM Syndrome Scale, should be used in every study center. Those researcher in clinical research should be relatively fixed. They should study and discuss the clinical research protocol and the manual of clinical research carefully. The way of recording and the standards of judgment should be unified.

#### Plans to promote participant retention and complete follow-up {18b}

A participant follow-up record form is set up for this study, and the follow-up of participants is managed by the clinical research coordinator (CRC). Clinical research coordinators record patients’ enrollment time, follow-up time, dose of drug, and remaining dose of drug at every follow-up time. Clinical research coordinators contact the participants to go to hospital on the follow-up day, and the research doctors evaluate the patient on the follow-up day and distribute drugs of the next phase. Every time the participants go to the hospital, they receive appropriate transportation subsidy. Before joining the project, the patient is promised to receive further treatment guidance from a specialist after the trial. If the subjects drop out of the trial or interrupt the trial, the data of the subjects is blinded with other subjects until the trial is completed. And the data of these subjects is statistically analyzed separately before uncovering blindness and finally are explained separately in the outcome part of this trial.

#### Data management {19}

The sponsor collects the paper version of case report forms (CRFs) of patient data for the proper storage as the study raw materials. All data are entered into the EDC, and any traces of entries, modifications, deletions etc. in the EDC are retained in the log showing who and when they were changed. Paper version data are input into the EDC system by two researchers in the sub-center, and double entry is adopted. The electronic data are backed up, and the backed-up electronic data are saved by another person. The sponsor checks the data again according to CRF to ensure the accuracy of the data. The sponsor arranges statisticians to complete the screening and randomization of research data. The paper-based CRF is sent to the sponsor in a safe and sealed way to ensure that they are not lost or leaked in the middle way. And all the paper-based CRF forms and the subjects’ information in EDC system are properly kept by the sponsor.

#### Confidentiality {27}

All patients’ data are kept confidential and not disclosed. Only study physicians granted authority by the sponsor have access to the EDC by account number and password and can only enter and review patient data at their site. The statistician and sponsor have access to the data for all participants. The sponsor checks the entered data regularly to ensure confidentiality. Patients’ data are stored in raw medical records at each hospital and anonymized stored in EDC for at least 5 years. Participants are allocated an individual trial identification number and participant’s details are stored on a secure database. Anonymized trial data cannot be shared with other researchers to enable international prospective meta-analyses.

#### Plans for collection, laboratory evaluation, and storage of biological specimens for genetic or molecular analysis in this trial/future use {33}

After signing an informed consent form, patients are given a baseline blood sample and retention urine sample, in which the blood routine, liver, and kidney function and urine routine are safety evaluation indicators. Meanwhile, a portion of blood samples are used for DNA methylation detection and a portion of urine samples are used for metabolomics detection. These blood and urine tests are performed again when the patient is out of the group.

Blood and urine samples are stored properly in −80°C and not be used by any other route than the study. Meanwhile, the existing as well as further sample specific studies will be conducted with ethics committee approval. If there are any remaining research specimens, biological specimens will be allowed to be used for future research in the case of reapplying for ethical approval.

### Statistical methods

#### Statistical methods for primary and secondary outcomes {20a}

Investigators establish the electronic case into the EXCEL form when making follow-up observations by interviewing the participants. Paper is entered into a database by those investigators in order to ensure data validity. All analyses are performed using SPSS software version 21.0. Differences are considered to be statistically significant for two-sided *P*<0.05. Comparisons between groups are conducted by using an analysis of covariance (ANCOVA). The significance of the cure rate, improvement rate, and unhealed rate differences between the groups are compared using ridit analysis and chi-square test.

#### Interim analyses {21b}

Security monitoring follows through the study at all times. Adverse events are reported immediately to the principal investigator, who is required to report them to the clinical trial monitoring committee within the prescribed period of time, depending on the severity. The clinical trial monitoring committee decide on a case by case basis whether to terminate the study.

No efficacy interim analysis is set up in this study.

#### Methods for additional analyses (e.g., subgroup analyses) {20b}

There will be no subgroup analysis based on gender for example or mile or moderate depression.

#### Methods in analysis to handle protocol non-adherence and any statistical methods to handle missing data {20c}

Participants with missing primary or secondary outcome data are excluded.

#### Plans to give access to the full protocol, participant level-data, and statistical code {31c}

This study protocol is registered with the registration number ISRCTN12746343 on the International Standard Randomized Controlled Trial (http://www.isrctn.com/ISRCTN12746343). The datasets analyzed during the current study are available from the corresponding author on reasonable request.

### Oversight and monitoring

#### Composition of the coordinating center and trial steering committee {5d}

There are several clinical study coordinators at each study center. Study coordinators play a role in coordination and study management. The trial steering committee (TSC) has an independent chair. In addition, the TSC has two public/patient group representatives, two clinical trials unit directors, statistician, and the principal investigator. If a member of the TSC withdraws during the course of the trial, we identify a replacement with a similar background. The members of TSC meet at the start of the trial and then weekly to review recruitment rates, protocol amendments, and any protocol deviations identified and may make recommendations to the sponsor regarding running the trial.

#### Composition of the data monitoring committee, its role, and reporting structure {21a}

The data monitoring committee is set up by the Beijing University of Chinese Medicine, and its members have no interest relationship with the sponsor. The members should have professional knowledge of medicine, pharmacy, health statistics, and be familiar with relevant laws and regulations of clinical trials. The committee monitors the eligibility, integrity, safety, and effectiveness of data in each research center through EDC system and question the problem data, which can be directly marked in EDC and generate a verification log. The modification traces of data by researchers in each center are also one of the monitoring contents. The data verification function of the EDC system is exclusive to the data monitoring committee, and the sponsor and researcher have no authority to carry out it. The committee also supervises protocol compliance, participant recruitment, and paper version clinical efficacy report every 2 weeks.

#### Adverse event reporting and harms {22}

1. Once an adverse event occurs, the investigator shall state to the subject that the subject is required to report truthfully the changes in his condition following the medication. Physicians are to avoid inducing questions.
2. While observing efficacy, pay close attention to observing adverse events or unanticipated toxic side effects (including symptoms, signs, laboratory tests), analyze the causes, make judgment, and follow-up observations and records.
3. For adverse events occurring during the study period, their symptoms, extent, time of appearance, duration, handling measures, experience, etc. should be recorded on a case report form, evaluated their relevance to the study drug, and recorded in detail by the investigator, signed, and dated.
4. When an adverse event is identified, the observing physician may decide whether to discontinue observation based on the condition, and cases who discontinue because of adverse effects should be followed up with a detailed record of the handling and outcome.
5. In the event of a serious adverse event in a study, the unit that assumes the responsibility for the clinical study must take immediate measures to protect the safety of subjects, must report to the project leader and the Research Center for clinical trials of drugs, should report within 24 h to the drug administration, the subject responsible unit, and the ethics committee. The investigator should also report to the data monitoring committee and relevant regulatory bodies as required indicating expectedness, seriousness, severity, and causality.
6. Investigator should sign and date on report. The subject responsible unit will guarantee reporting procedures that meet all legal and regulatory requirements.
7. When urgent breaking of blinding is required for a serious adverse event to occur in a clinical study, the blinding should be broken jointly by the project leader, investigator, clinical monitor.

#### Frequency and plans for auditing trial conduct {23}

The sponsor and the study center meet weekly to ensure that the study is being conducted in accordance with the study protocol.

#### Plans for communicating important protocol amendments to relevant parties (e.g., trial participants, ethical committees) {25}

If the study protocol is amended, it must be approved by the ethics committee of the Beijing University of Chinese Medicine, and patients are also provided written informed consent to inform the modification. Sponsor and funder are notified first. Then, the principle investigator notifies the centers and a copy of the revised protocol is sent to the principle investigator to add to the Investigator Site File. Any deviations from the protocol are fully documented using a breach report form. The protocol also is updated in the clinical trial registry.

#### Dissemination plans {31a}

The results of this study will be published in a scientific journal.

## Discussion

Depression is a common affective mental disorder, which is associated with a high burden of disease. Research suggests that chronic diseases, genetic factors, social, and environmental factors can induce depression [22]. The common therapy for depression is antidepressants. Due to the side effects of drugs for depression, the compliance of patients is rather poor and then affect the drug efficacy [23]. Therefore, it is urgent to find safe and no side-effect treatment. A number of patients with depression would prefer traditional Chinese medicine over pharmacotherapy when our study demonstrates the effectiveness and safety of traditional Chinese medicine to treat depression. This will be especially relevant for patients who do not respond to medical therapy or who experience adverse side effects to drug therapy.

According to traditional Chinese medicine theory, liver stagnation and spleen deficiency syndrome is one of the main syndromes of depression. Especially due to people’s unhealthy eating habits and long-term emotional stress from life and work in modern society, the symptoms of the liver stagnation and spleen deficiency are very common. In clinical practice, the syndrome of liver stagnation and spleen deficiency has become a high incidence and common syndrome. Xiaoyao pills has good effect of soothing the stagnated liver and strengthening the spleen. In our study, TCM syndrome efficacy evaluation is added in order to determine more suitable patient populations to take Xiaoyao pills and optimize indications.

The potential mechanism of Xiaoyao pills to treat depression mainly focused on animal experiments [24–28]. There is little evidence about the potential mechanism about Xiaoyao pills to treat depression in clinical studies. Therefore, in addition to the efficacy evaluation of Xiaoyao pills, the action mechanism of Xiaoyao pills from the perspective of transcriptomics and DNA methylation sequencing will also be studied in our trial.

However, our study has limitation. The limitation concerns the fact that we will only recruit mild to moderate depression patients with syndrome of liver stagnation and spleen deficiency in order to ensure the curative effect. Effectiveness of TCM for different types of depression is not evaluated.

Therefore, a clinical trial about traditional Chinese medicine to treat different types of depression are still needed.

### Trial status

The trial is currently in the recruitment phase. *This study protocol is version 3 made on June 1, 2020. Recruitment commenced in March 2020 at Peking University Sixth Hospital and is expected to be completed in December 2021.*

## Abbreviations

DSM-5: Diagnostic Statistics Manual of Mental Disorders
HAMD: Hamilton Depression Scale
TCM: Traditional Chinese medicine
LC-M: Liquid chromatograph-mass spectrometer
GO: Gene ontology
KEGG: Kyoto Encyclopedia of Genes and Genomes
AEs: Adverse events
CRF: Case report forms
CRC: Clinical research coordinator
TSC: Trial Steering Committee (TSC)
ANCOVA: Analysis of covariance
EDC: Electronic data capture
